# Supplementary material for: Exchange-biased topological transverse thermoelectric effects in a Kagome ferrimagnet
Source: Nat Commun. 2022 Mar 1;13:1091. doi: 10.1038/s41467-022-28733-7 (PMC8888656; doi:10.1038/s41467-022-28733-7)
Supplement: Supplementary file 1 — Supplementary Information [file 41467_2022_28733_MOESM1_ESM.pdf]

Supplementary Information of "**Exchange-biased topological transverse thermoelectric effects in a Kagome ferrimagnet**"

Heda Zhang[1,#], Jahyun Koo[2,#], Chunqiang Xu[1,3,#], Milos Sretenovic[1], Binghai Yan[2], and Xianglin Ke[1*]

[1]*Department of Physics and Astronomy, Michigan State University, East Lansing, Michigan 48824-2320, USA*

[2] *Department of Condensed Matter Physics, Weizmann Institute of Science, Rehovot, Israel*

[3] *School of Physics, Southeast University, Nanjing 211189, China*

1. Seebeck coefficient of $TbMn_6Sn_6$

Supplementary Figure 1 shows the temperature dependence of the Seebeck coefficient S_{xx} of $TbMn_6Sn_6$. S_{xx} decreases monotonically with temperature and shows a sign change around 25 K. The sign change of S_{xx} has also be observed in Fe_3Sn_2, which likely results from the competing contributions from electron diffusion and phonon drag [1].

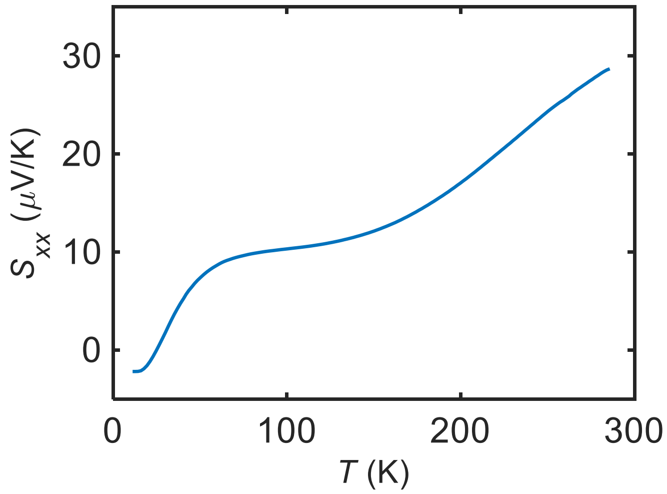

Supplementary Figure 1. Seebeck coefficient of $TbMn_6Sn_6$ measured as a function of temperature.

2. Sign convention of transverse transport measurements

Here we elaborate the sign convention of transverse transport measurements. For this purpose, Supplementary Figure 2 shows the schematics of experimental device.

i) ρ_{xx}, κ_{xx} and S_{xx}: for ρ_{xx}, κ_{xx}, the sign is always positive. For S_{xx}, the sign is defined as $S_{xx} = -\frac{\Delta V_{xx}}{\Delta T_{xx}} = -\frac{v_2 - v_1}{|\Delta T_{xx}|}$, which is widely used.

ii) σ_{xy}, κ_{xy} : for $\sigma_{xy} = \frac{-\rho_{xy}}{\rho_{xx}^2+\rho_{yx}^2} = \frac{\rho_{yx}}{\rho_{xx}^2+\rho_{yx}^2}$, where σ_{xy} and ρ_{yx} shares the same sign. A schematic figure is shown in Supplementary Fig. 2(a). We define $\rho_{yx} = \frac{V_{yx}}{I} = \frac{V_3 - V_2}{I}$, where I is the magnitude of the electric current passing through the sample. Note that the magnetic field direction, electric current direction and Hall electric field direction define a right-handed coordinate system [i.e., $\vec{E}_y // (\vec{H} \times \vec{I})$]. This definition is the same as the definition used by L. Ye et al [2], as illustrated by the inset in Figure 2(c) of this reference. For κ_{xy}, we used the same definition as the one used in σ_{xy}. That is, we replace σ_{xy} with κ_{xy}, ρ_{yx} with w_{yx}. V_i with T_i, and I with I_{heat}.

iii) For S_{xy}: we used the same convention as described by T. Liang et al [3]. As shown in Supplementary Fig. 2(b), the magnetic field direction, heat current direction and Nernst electric field direction define a right-handed coordinate system [i.e., $\vec{E}_y // (\vec{H} \times \vec{I}_{heat})$]. The Nernst coefficient's sign is defined by $S_{xy} = \frac{(V_3 - V_2)/w}{|\Delta T_{xx}|/l}$.

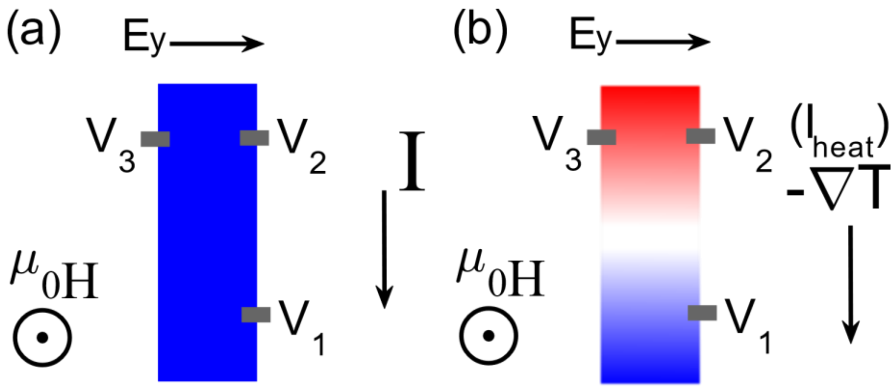

Supplementary Figure 2. Sign definition for Hall (a) and Nernst signals in our experimental set-up.

3. **Experimental raw data of transverse transport measurements, data processing, and supplemental calculation data**

Supplementary Figures S3 -S5 present the raw measurement data of σ_{xy}, S_{xy} and κ_{xy}. We see that both normal and anomalous components are present in these data. The red curves represent the fitting results as to be described next. At higher temperatures, only a linear function is fitted through the high field region to subtract the normal component.

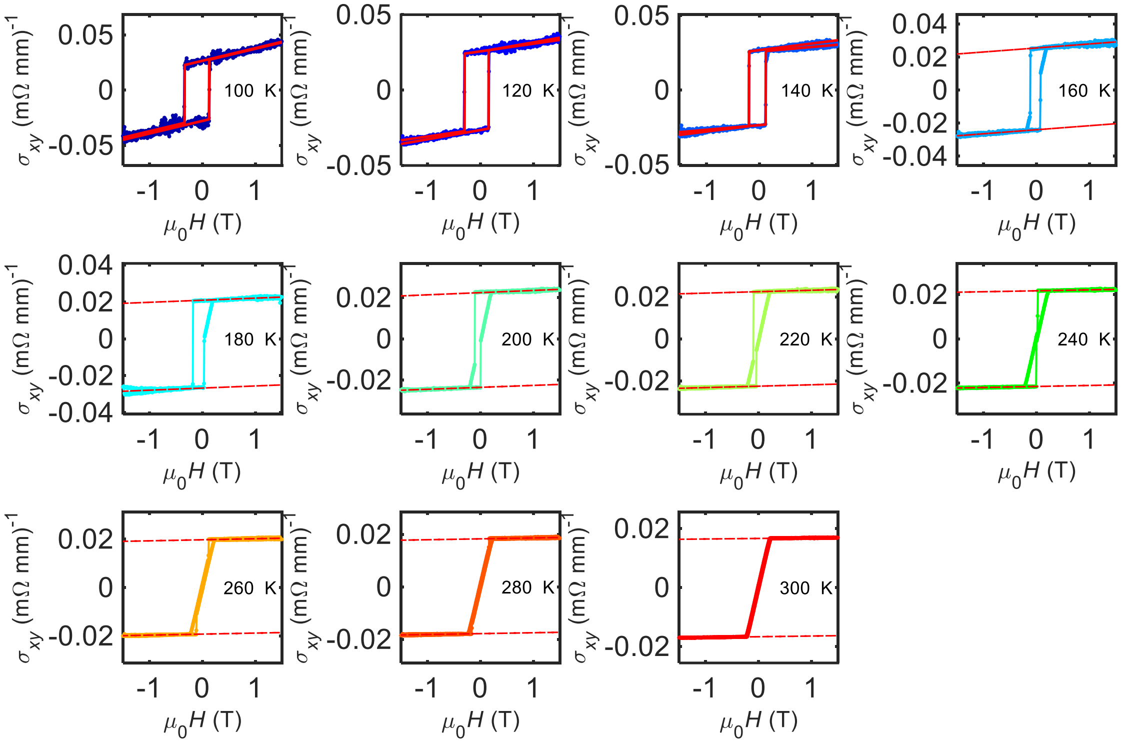

Supplementary Figure 3. Raw data and fitting results for electric Hall conductivity σ_{xy} measured at various temperatures.

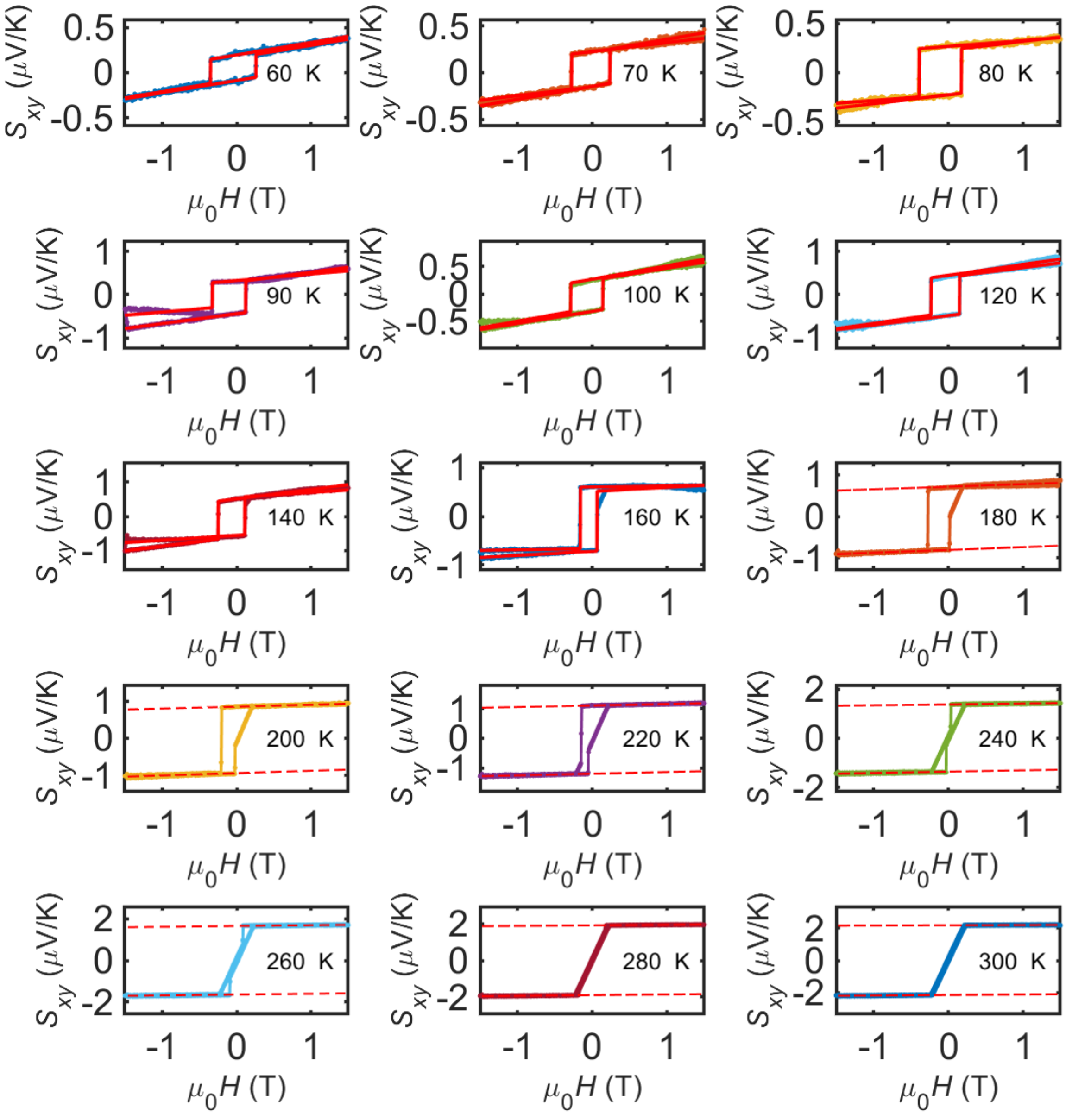

Supplementary Figure 4. Raw data and fitting results for Nernst coefficient S_{xy} measured at various temperatures.

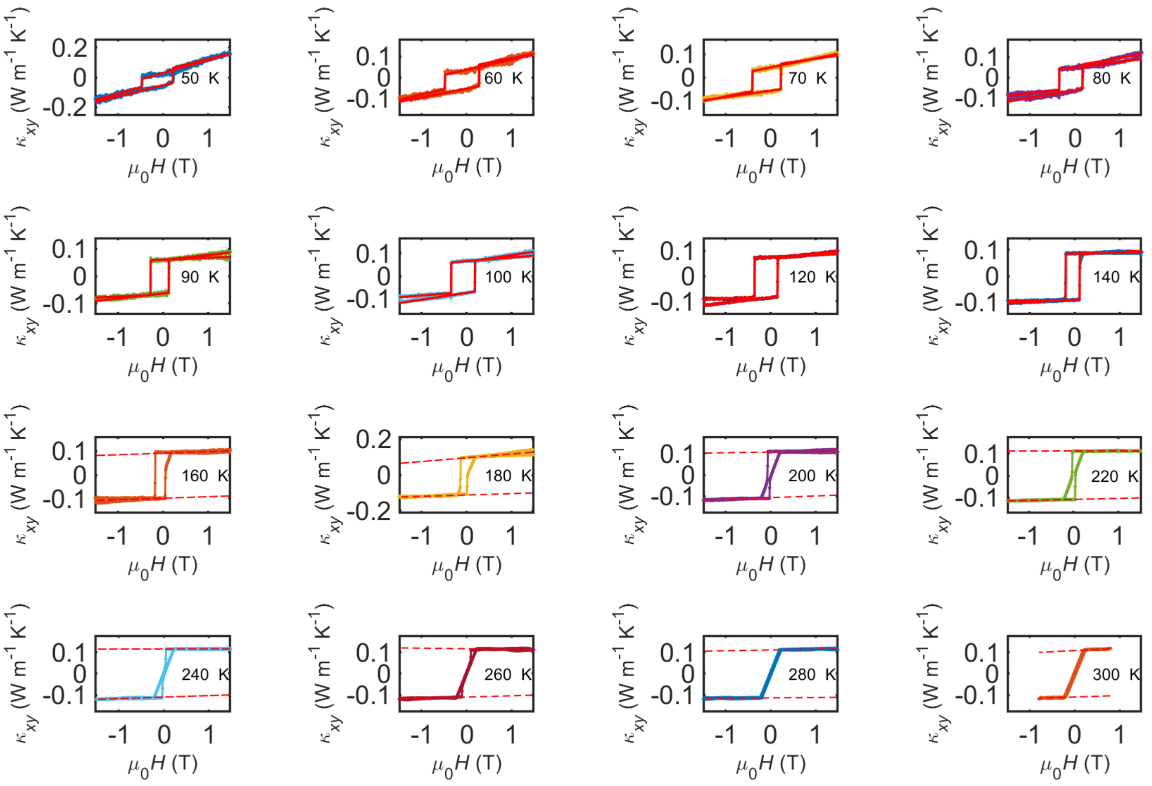

Supplementary Figure 5. Raw data and fitting results for thermal Hall conductivity κ_{xy} measured at various temperatures.

We extracted the anomalous components following the procedure described here. For lower temperature data (e.g., 100 K), we started by fitting the sweep-up curve and sweep-down curve using the formula: $y = A * Heaviside(x - x_0) + B * x + C$. By allowing different x_0 values for sweep-up and sweep-down curves, we obtained the fitting results shown by the red curves in Supplementary Fig. 6(a). We then can get rid of the linear term in our data and obtain the anomalous contributions S_{xy}^{A} as shown in Supplementary Fig. 6(c). At higher temperatures (e.g., 240 K shown in Supplementary Fig. 6(b)) where both the exchange-bias feature and the normal component are weak, we directly fit the high field linear term to obtain the anomalous component as shown in Supplementary Fig. 6(d). After obtaining the anomalous components, the

anti-symmetrizing procedure goes as follow: the blue curves show the processed data described above, the orange curves show the same data but being reflect by $x \rightarrow -x$.

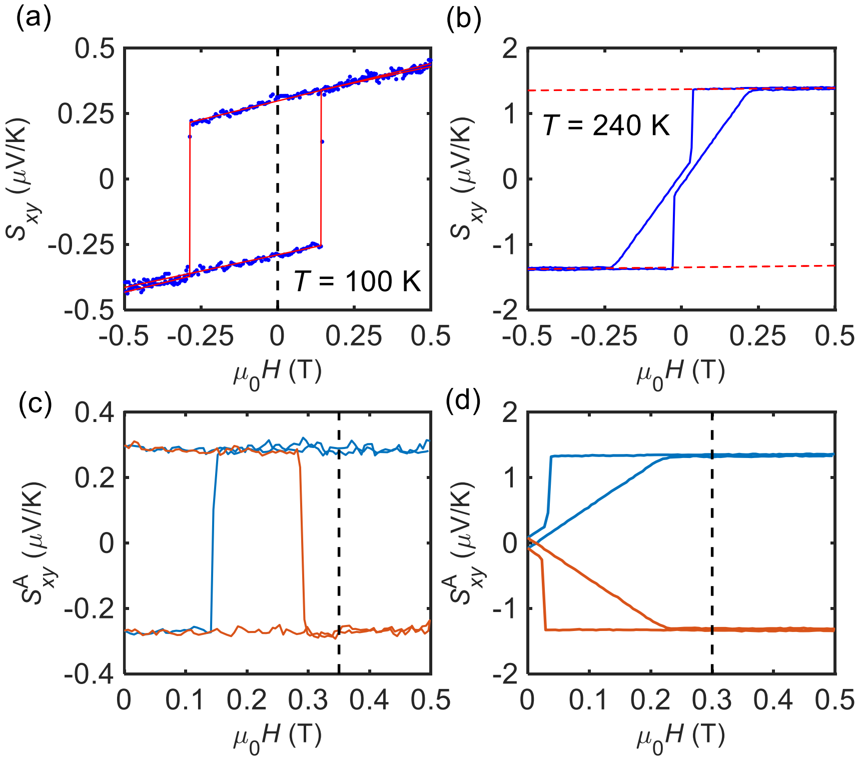

Supplementary Figure 6. The Nernst measurement data at two typical temperatures 100 K (a) and 240 K (b). The red solid line in (a) represents a fitting to the data as described above, the red dash line in (b) represents the linear fitting to normal Nernst signal. The anomalous Nernst signal for 100 K and 240 K are presented in (c) and (d) respectively. The blue curve indicates $S_{xy}^{A}(H)$ whereas the red curve indicates $S_{xy}^{A}(-H)$.

We would like to point out that our thermoelectric and thermal transport measurements were done in an adiabatic manner. The rational is as follows. During our data analysis, we were aware of the transverse ‘Seebeck’ voltage induced by the Righi-Leduc effect (thermal Hall effect) in our set-up. We determined the relative importance of isothermal Nernst effect and the transverse ‘Seebeck’ voltage in an adiabatic process by comparing the anomalous Nernst angle (θ_{ANE}) and the anomalous thermal Hall angle (θ_{ATHE}). Our rational behind this goes as follows:

the total transverse voltage is composed of two contributors in an adiabatic setting, $V_{xy}^{adb} = S_{xy}^{iso}\Delta T_{xx} - S_{yy}\Delta T_{yx}$ (The minus sign here reflects the Seebeck and thermal hall sign definition described previously). Dividing the two sides of the equation by ΔT_{xx} (here, isotropic Seebeck coefficients is assumed, i.e., $S_{xx} = S_{yy}$), one obtains $S_{xy}^{adb} = \left(\theta_{ANE}^{iso} - \frac{l}{w}\theta_{ATHE}\right) * S_{xx}$, where l = 1.11 mm and w = 1.15 mm are the length and width of contact leads for the transport data presented in this study. Supplementary Figure 7 shows the measured adiabatic ANE angle (θ_{ANE}^{adb}) and the θ_{ANE}. We can see that there is nearly one order of difference between the adiabatic signal and the signal induced by Righi-Leduc effect. As a result, the measured S_{xy}^{adb} is mainly dominated by the isothermal S_{xy}^{iso}.

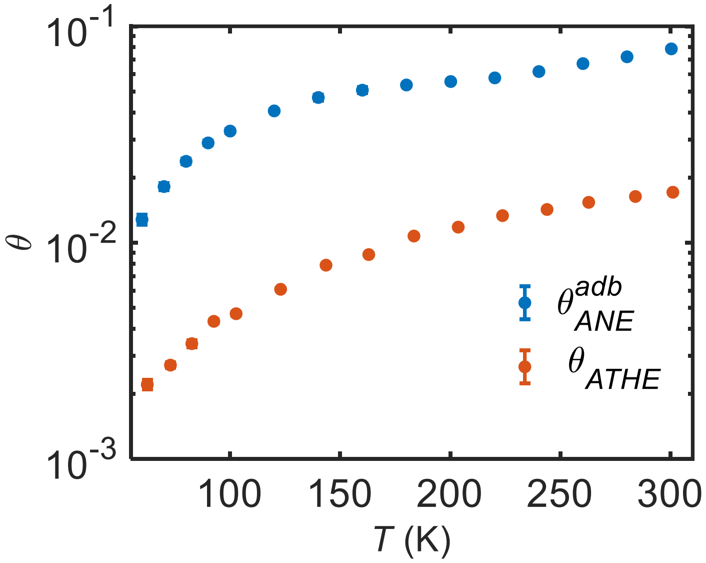

Supplementary Figure 7. Temperature dependence of anomalous Hall angle (θ_{ATHE}) and anomalous Nernst (adiabatic) angle (θ_{ANE}^{adb}). There is nearly an order of magnitude difference of these two Hall angles in the whole temperature range measured.

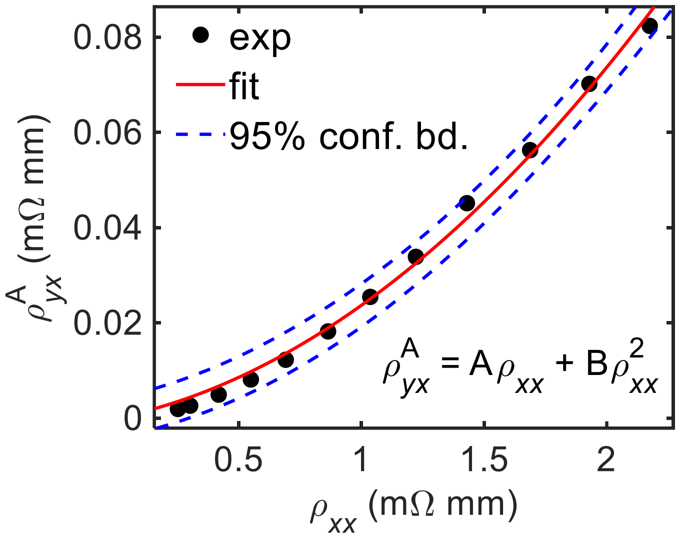

Supplementary Figure 8. The fitting result to the measurement anomalous Hall resistivity using the formular $\rho_{yx}^{A} = \rho_{yx}^{sk} + \rho_{yx}^{int} = A\rho_{xx} + B\rho_{xx}^{2}$. Experiment data are shown as black markers. The red curve represents the fit and the blue curve represents the 95% confidence bound. The fitting parameters are $A = 0.01055 \pm 0.0035$, $B = 0.01314 \pm 0.00205$ $(\mathrm{m\Omega\ mm})^{-1}$.

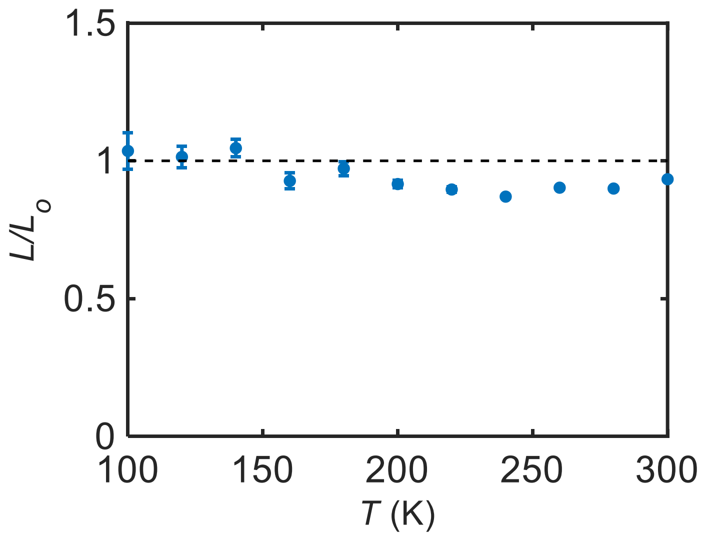

Supplementary Figure 9. The ratio between anomalous thermal Hall conductivity and anomalous Hall conductivity scaled by temperature $\left(\frac{\kappa_{xy}^{A}}{\sigma_{xy}^{A}T}\right)$ in units of Lorenz number L_0 $(2.44 \times 10^{-8} V^2 K^{-2})$, indicating that the ‘anomalous’ Wiedemann–Franz law is reasonably well obeyed in the temperature range measured.

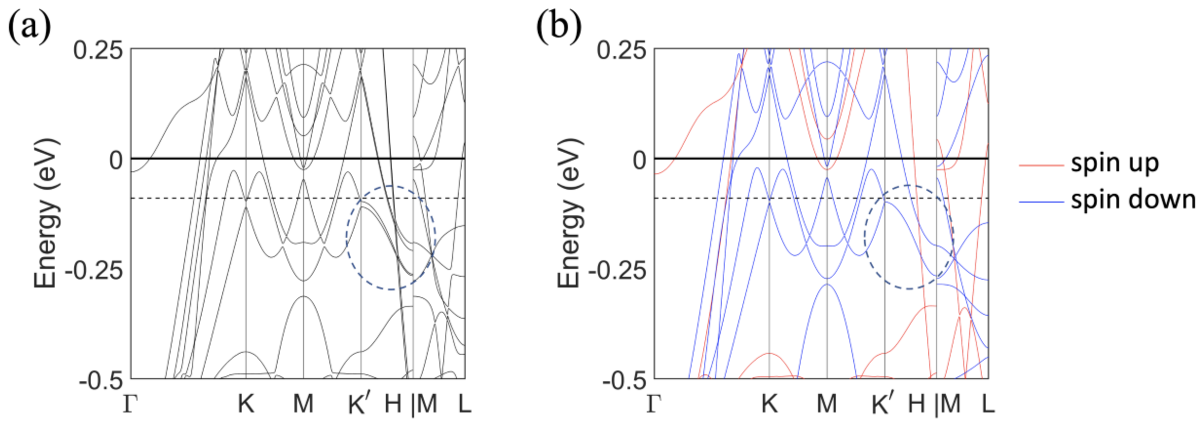

Supplementary Figure 10. The calculated band structure with (a) spin-orbit coupling and (b) without spin-orbit coupling. There are nodal lines along K(K')-H near the chemical potential (dashed horizontal line). The spin-orbit coupling lifts off the degeneracy at the K (K') point and along the K(K')-H line. Thus, the massive Dirac gap at K(K') originates from gapping the nodal line. The nodal line scenario is caused by symmetry-breaking, independent from interaction or Tb *f* electrons.

4. Supplemental data of the exchange-bias behavior

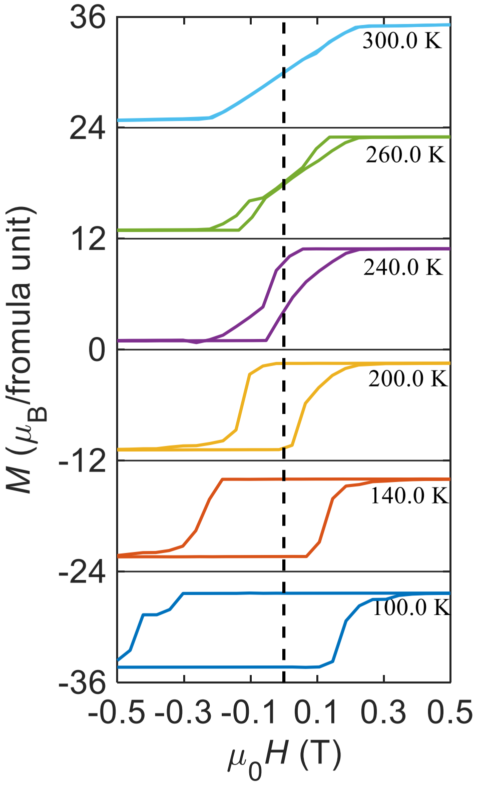

Supplementary Figure S11. Magnetic field dependence of magnetization. These data were taken after the sample was initially cooled down to 100 K with 1.5 T magnetic field but without the subsequent warming up and cooling down processes for the measurements at each temperature.

Supplementary Figure 12 presents more comprehensive $M(H)$ datasets. Distinct from Supplementary Fig. 11, for Supplementary Fig. 12, Prior to each $M(H)$ measurement, the sample was warmed up to 340 K and then cooled down to the measurement temperature with an applied field of 0.5 T.

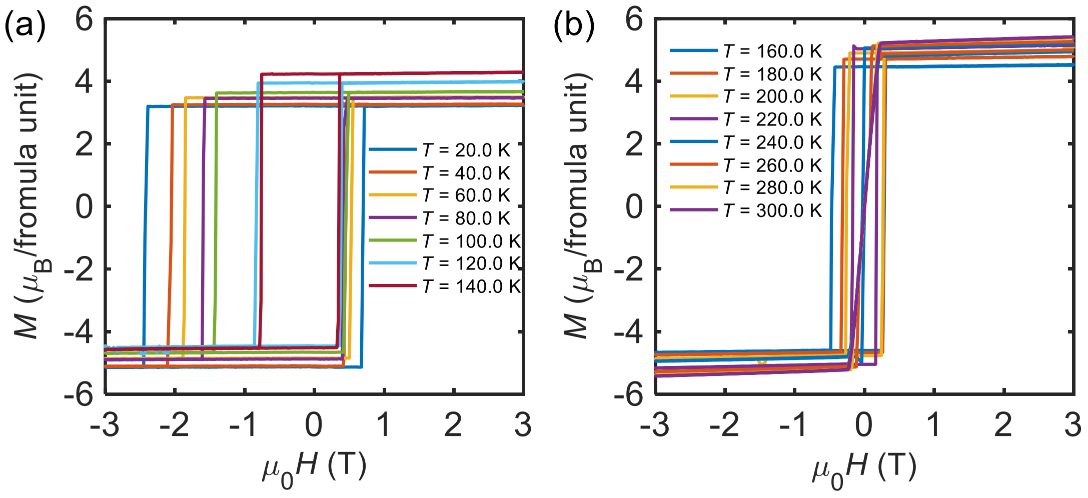

Supplementary Figure 12. The magnetization data of $TbMn_6Sn_6$ measured at various temperatures.

5. Competing interlayer couplings in $TbMn_6Sn_6$

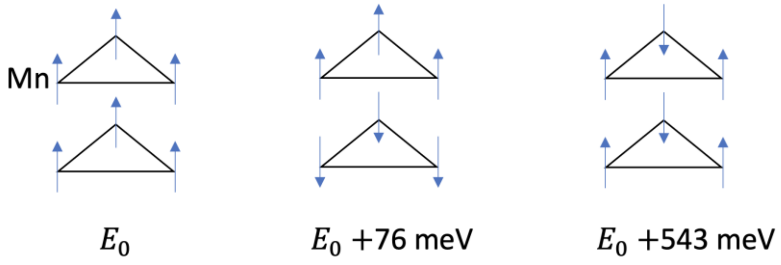

Supplementary Figure 13. The schematic magnetic structures of Mn atoms and their energies in DFT calculations. The ground state is ferromagnetic coupling for inter- and intra-layer Mn sites. Note that the Tb spins always align antiparallel to Mn spins in the neighboring layers. The interlayer exchange coupling is much weaker compared to the intralayer one and it is more likely to exhibit antiferromagnetic interlayer coupling than intralayer coupling.

6. ac susceptibility data of $TbMn_6Sn_6$

Supplementary Figure 14 shows the ac magnetic susceptibility as functions of temperature measured at various frequencies with an excitation field of 5 Oe. Both the real (χ') and imaginary (χ'') susceptibility exhibit frequency dependence. We fit the ac susceptibility data using a conventional critical slowing-down power-law relaxation $\tau = \tau_0 \left(\frac{T_f}{T_g} - 1\right)^{-zv}$, where $\tau = \frac{1}{2\pi f}$ with f being the measurement frequency, τ_0 the spin flipping relaxation time of the fluctuating entities, T_g the static freezing temperature, T_f the temperature of the χ'' maximum at each measurement frequency, and zv the dynamic exponent. The result is shown in the inset of Supplementary Fig. 14. Based on the fitting, we find for T_g = 175 K, $\tau_0 = 5.13 * 10^{-7} s$, and $zv = 2.46$, suggesting the formation of a cluster spin glass state instead of conventional spin glass due to individual spin randomness. For conventional spin glass, $\tau_0 \sim 10^{-13} - 10^{-10}\ s$ and $zv \sim 4 - 13$ (Ref. [4]). In addition, χ'' peaks around 180 K, coinciding the onset of the exchange-bias feature shown in the

Fig. 4(a,c,e,f) in the main text. This implies that the cluster spin glass phase coexisting with the bulk ferrimagnetic phase is the key to the exchange-bias phenomena observed in $TbMn_6Sn_6$.

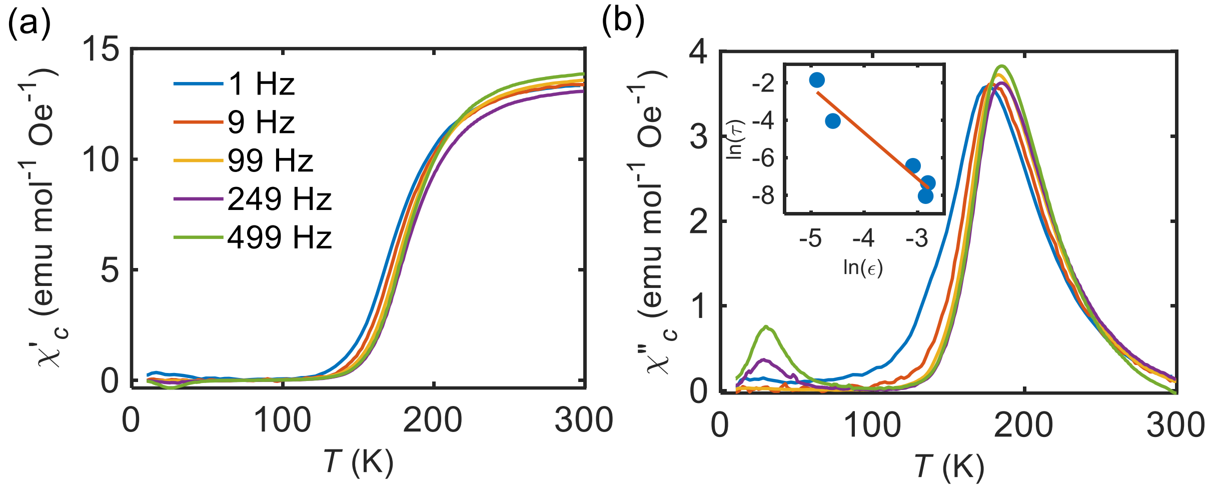

Supplementary Figure 14. The real χ' (a) and imaginary χ'' (b) part of the ac magnetic susceptibility of $TbMn_6Sn_6$. Inset shows the dynamical scaling of τ vs. the reduced temperature $\epsilon = \frac{T_f}{T_g} - 1$. The solid line is the fitting as described in the text.

References

1 Zhang, H., Xu, C. Q. & Ke, X. Topological Nernst effect, anomalous Nernst effect, and anomalous thermal Hall effect in the Dirac semimetal Fe_3Sn_2. *Physical Review B* **103**, L201101, doi:10.1103/PhysRevB.103.L201101 (2021).

2 Ye, L., Kang, M., Liu, J., von Cube, F., Wicker, C. R., Suzuki, T., Jozwiak, C., Bostwick, A., Rotenberg, E., Bell, D. C., Fu, L., Comin, R. & Checkelsky, J. G. Massive Dirac fermions in a ferromagnetic kagome metal. *Nature* **555**, 638-642, doi:10.1038/nature25987 (2018).

3 Liang, T., Lin, J., Gibson, Q., Gao, T., Hirschberger, M., Liu, M., Cava, R. J. & Ong, N. P. Anomalous Nernst Effect in the Dirac Semimetal Cd_3As_2. *Physical Review Letters* **118**, 136601, doi:10.1103/PhysRevLett.118.136601 (2017).

4 Luo, Q., Zhao, D. Q., Pan, M. X. & Wang, W. H. Critical and slow dynamics in a bulk metallic glass exhibiting strong random magnetic anisotropy. *Applied Physics Letters* **92**, 011923, doi:10.1063/1.2827198 (2008).
